# Mesenchymal Stem Cells from Fetal Heart Attenuate Myocardial Injury after Infarction: An *In Vivo* Serial Pinhole Gated SPECT-CT Study in Rats

**DOI:** 10.1371/journal.pone.0100982

**Published:** 2014-06-27

**Authors:** Venkata Naga Srikanth Garikipati, Sachin Jadhav, Lily Pal, Prem Prakash, Madhu Dikshit, Soniya Nityanand

**Affiliations:** 1 Stem Cell Research Facility, Department of Hematology, Sanjay Gandhi Post-Graduate Institute of Medical Sciences, Lucknow, India; 2 Department of Pathology, Sanjay Gandhi Post-Graduate Institute of Medical Sciences, Lucknow, India; 3 Cardio-vascular unit, Division of Pharmacology, Central Drug Research Institute, Lucknow, India; University of Kansas Medical Center, United States of America

## Abstract

Mesenchymal stem cells (MSC) have emerged as a potential stem cell type for cardiac regeneration after myocardial infarction (MI). Recently, we isolated and characterized mesenchymal stem cells derived from rat fetal heart (fC-MSC), which exhibited potential to differentiate into cardiomyocytes, endothelial cells and smooth muscle cells *in* *vitro*. In the present study, we investigated the therapeutic efficacy of intravenously injected fC-MSC in a rat model of MI using multi-pinhole gated SPECT-CT system. fC-MSC were isolated from the hearts of Sprague Dawley (SD) rat fetuses at gestation day 16 and expanded *ex* *vivo*. One week after induction of MI, 2×10^6^ fC-MSC labeled with PKH26 dye (n = 6) or saline alone (n = 6) were injected through the tail vein of the rats. Initial *in* *vivo* tracking of 99mTc-labeled fC-MSC revealed a focal uptake of cells in the anterior mid-ventricular region of the heart. At 4 weeks of fC-MSC administration, the cells labeled with PKH26 were located in abundance in infarct/peri-infarct region and the fC-MSC treated hearts showed a significant increase in left ventricular ejection fraction and a significant decrease in the end diastolic volume, end systolic volume and left ventricular myo-mass in comparison to the saline treated group. In addition, fC-MSC treated hearts had a significantly better myocardial perfusion and attenuation in the infarct size, in comparison to the saline treated hearts. The engrafted PKH26-fC-MSC expressed cardiac troponin T, endothelial CD31 and smooth muscle sm-MHC, suggesting their differentiation into all major cells of cardiovascular lineage. The fC-MSC treated hearts demonstrated an up-regulation of cardio-protective growth factors, anti-fibrotic and anti-apoptotic molecules, highlighting that the observed left ventricular functional recovery may be due to secretion of paracrine factors by fC-MSC. Taken together, our results suggest that fC-MSC therapy may be a new therapeutic strategy for MI and multi-pinhole gated SPECT-CT system may be a useful tool to evaluate cardiac perfusion, function and cell tracking after stem cell therapy in acute myocardial injury setting.

## Introduction

Cellular cardiomyoplasty has emerged as a potential therapeutic strategy for patients with acute myocardial infarction (MI). MI results in loss of cardiomyocytes, ventricular remodeling, scar formation, fibrosis and subsequently heart failure [1]. The ultimate goal of any regenerative therapy for ischemic myocardium is to regenerate lost cardiomyocytes and facilitate cardiovascular neovascularization, in order to lead to clinical improvement in cardiac functions. An array of adult stem cell types including skeletal myoblasts, bone marrow derived stem cells, endothelial progenitor as well as cardiac stem cells have been shown to lead to functional benefit in animal models of infarction [2]–[5], but clinical trials have generated mixed results [6]–[8]. Hence, a search for a novel stem cell type that is capable of restoring cardiac function is of paramount importance.

Mesenchymal stem cells (MSC) due to their characteristic properties such as ease of isolation, extensive *ex* *vivo* expansion capacity and multi-lineage differentiation potential are considered to be one of the potential stem cells for cardiac repair and regeneration after MI in both experimental animals [9], and clinical studies [10]. Although originally identified in bone marrow, MSC have also been isolated from many adult organs as well as fetal-stage tissues [11]. Recently it has been suggested that the developmental stage of donor tissues not only affects the ability of MSC to differentiate into cardiomyocyte, but also their capacity to undergo smooth muscle and endothelial differentiation [12]. Moreover, it has been shown that tissue specific MSC possess unique properties with inherent potential of differentiation in to cell lineages of their tissue of origin [13]. In this context, we recently isolated and characterized MSC derived from rat fetal heart and described these cells as fetal cardiac mesenchymal stem cells (fC-MSC). They exhibited the potential to differentiate in to cardiomyocytes, endothelial cells and smooth muscle cells over successive passages, while maintaining expression of TERT and a normal karyotype [14].

Because of the enormous potential of cardiac stem cell therapy, it is being rapidly translated into clinical trials, and thus has left many issues unresolved, and emphasizes the need for concurrent techniques that provide more insights in to the mechanisms involved [15]. Molecular imaging is likely to play an important role in the better understanding of the fate of stem cells and their contribution in recovery of cardiac function [16]. Myocardial gated SPECT/CT is widely accepted as a gold standard for clinical measurement of cardiac functions [17]. With use of pinhole collimators and the advances in data processing, gated SPECT/CT has recently been adapted for small animal cardiovascular molecular imaging [18].

Taken together, we designed the present study to investigate the therapeutic efficacy of intravenously injected fC-MSC in a clinically most relevant rat model of MI (cardiac ischemia-reperfusion (IR) injury), using multi-pinhole gated SPECT/CT system. We also sought the cellular and molecular mechanisms underlying the beneficial effects of fC-MSC therapy.

## Materials and Methods

### Animals

Adult Sprague-Dawley (SD) rats, aged 8–12 weeks, weighing 180–250 g, were used in all experiments. Animals were housed at a constant temperature and humidity, with a 12∶12-h light-dark cycle, and had free access to a standard diet and water. All the procedures were performed as per guidelines of Institutional Animal Ethics Committee and Committee for Purpose of Control and Supervision of Experiments on Animals (CPCSEA), India. The Committee on the Ethics of Animal Experiments of Sanjay Gandhi Post Graduate Institute of Medical Sciences, Lucknow, India, approved the protocol.

### Isolation, Culture and Characterization of rat fC-MSC

fC-MSC were isolated and cultured from the hearts of SD rat fetuses at gestation day 16, in accordance with the methods described previously [14]. In brief, fetal hearts (n = 10) were minced, digested with 1 mg/mL collagenase type-IV (Worthington Biochemical, USA) and cultured in 25 cm^2^ tissue culture flasks (Becton, Dickinson; USA) using complete culture medium consisting of α-MEM medium, 2 mg/mL of Glutamax (Gibco-Invitrogen), 16.5% fetal bovine serum (Hyclone, USA) and bacteriostatic level of penicillin-streptomycin (Gibco-Invitrogen). The MSC characteristics of the cells between 3^rd^–5^th^ passages were confirmed by flow cytometric positivity for CD29, CD44, CD73, CD90 and CD105 and negativity for CD31, CD45 and MHC-II or isotype-identical antibodies (IgG) were used as negative controls and differentiation into adipogenic and osteogenic cells using induction kits (Chemicon, USA).

### Rat model of MI

The left anterior descending (LAD) coronary artery of male SD rats (n = 12) was occluded using the method previously described [19], with slight modifications. In brief, all rats were anesthetized with 80 mg/kg ketamine and 10 mg/kg xylazine, injected intra-peritoneally, and then mechanically ventilated. The heart was exposed through a left thoracotomy at the 3^rd^–4^th^ intercostal space, and the left anterior descending coronary artery (LAD) was ligated with a 6–0 polyester suture and reperfused after 30 min of ligature.

On day 7 of MI induction, rats were randomized into two groups: (i) fC-MSC group (n = 6), and (ii) saline group (n = 6). A total of 2×10^6^ fC-MSC labeled with PKH26 dye or saline alone (150 µL each) were injected intravenously through tail vein in the two groups, respectively. In 06 additional SD rats, MI was induced and after 1 week of induction of MI, fC-MSC labeled with Tc99-HMPAO were injected and tracked after 6 h of injection.

The ischemic area and the left ventricle (LV) functions were monitored in all treated and untreated animals by serial 99Tc-sestamibi pinhole gated SPECT performed before fC-MSC therapy (1 week post MI induction) and 4 weeks after fC-MSC therapy. The animals were sacrificed 4 weeks after fC-MSC therapy, and the hearts were removed, rinsed and used to perform the assays described below.

### Cell Labeling

For initial tracking of fC-MSC after 6 h of injection, the cultured cells were trypsinized and incubated in a concentration of 2×10^6^ cells per mL at 37°C with 2 mCi of Tc-99 with HMPAO linker for a 10-min period, and then the labeling process was stopped by a 5-min centrifugation at 950 g. This 10-min incubation period was found to result in sufficient labeling efficiency (74%) without significant deterioration of cell viability (92%). The 99 m Tc-HMPAO labeled cells were conditioned in an insulin syringe (2×10^6^ cells per 50 µL), and a single injection was performed through tail vein.

For tracking of the cells 4 weeks after injection, fC-MSC were labeled with PKH26 (Sigma Aldrich, USA, kind gift from Dr Yucheng Dai, Molecular Medicine and Stem Cell Center of Second Affiliated Hospital of Jiangxi Medical Collage, Nanchang, PR China) according to manufacturer’s protocol. In brief, 2×10^6^ fC-MSC were taken in a 15 mL cone-shaped tube, washed with 10 mL PBS, centrifuged (200×g, 15 minutes) and a 25 µL of cell pellet was obtained. To the pellet, 1 mL of diluent C and 4×10^−6^ M of PKH26 staining reagent was added to obtain a final volume of 2 mL of 2×10^6^ fC-MSC and incubated at room temperature for 5 min. The same amount of 10% FBS DMEM (2 mL) was used to stop the staining reaction. Labeling efficiency was 95% as validated by observation of cells in fluorescence microscope. Cells were conditioned to an insulin syringe (2×10^6^ per 150 µL) to inject in animals.

### Pinhole Gated SPECT Acquisitions

The animals were sedated by intraperitoneal injection of 10 mg/kg xylazine and 80 mg/kg ketamine. 99mTc-sestamibi (2 mCi in a 0.3- to 0.5 mL volume) was injected intravenously 40–60 min before starting gated SPECT acquisition. During the acquisition, the animals were kept in prone position and were connected to a standard electrocardiogram monitor by 3 electrodes placed on the inner surfaces of limb extremities. Pinhole gated SPECT acquisitions were performed using a dual head γ-camera (Bioscan, USA) equipped with a 3-mm pinhole collimator (195-mm focal length; 43-mm radius of rotation). A number of 24 projections of 60 seconds per step were acquired on a 360° rotation and with 8 frames per cardiac cycle. Additional acquisition parameters were as follows: 64×64 matrix, 2.0 zoom, 126- to 154-keV energy window, beat acceptance window set to ±20% of averaged R-R interval. Total acquisition time was 24 min.

### Reconstruction and Analysis of Pinhole Gated SPECT Images

Using the HISPECT-NG software myocardial gated SPECT images were reconstructed. FlowQuant software (Ottawa Heart Institute, Canada) was used to generate myocardial perfusion polar maps and to determine LV end-diastolic volume (EDV) and end-systolic volume (ESV), as well as LV ejection fraction (EF) on the contiguous gated short-axis slices obtained from serial 99mTc-sestamibi gated SPECT. Further, polar maps were used to quantify the viable myocardium using the threshold value of 50% uptake of 99mTc-sestamibi [20].

### Immunohistochemistry

Heart tissues fixed in 4% paraformaldehyde were sectioned (5 µm) and incubated with antibodies specific for the detection of the following proteins; Troponin-T (1∶500; Serotec, UK), CD31 (1∶500; Santa Cruz), sm-MHC (1∶1000, Serotec), in 5% normal sheep serum overnight at 4°C. Next day, after washing with PBS, the sections were incubated with fluorochrome tagged respective secondary antibodies for 1 hour in dark. The sections were counterstained with Hoechst 33258 (Molecular probes) nuclear stain and mounted in antifade mountant. Heart sections stained with non-immune serum or IgG instead of the primary antibodies was used as negative control. The pictures were taken using fluorescent microscope (Nikon 80i, Japan).

### Real Time PCR

Heart tissues were harvested at 4 weeks post fC-MSC therapy, pulverized in liquid nitrogen, and homogenized in 1 mL Trizol reagent (Invitrogen). Total RNA was extracted with 0.2 ml of chloroform and precipitated with 0.5 ml 80% (vol/vol) isopropanol. After the removal of the supernatant, the RNA pellet was washed with 70% (vol/vol) ethanol, air-dried, and dissolved in DNase RNase-free water. Complementary DNA was synthesized from 1 µg of RNA using First-Strand cDNA Synthesis Kit (USB, USA) at 44°C for 60 minutes, 92°C for 10 minutes. Real-time PCR with relative quantification of target gene copy numbers in relation to β-actin transcripts was carried out using the following primers given in Table 1. Relative fold expression values were determined applying the ΔΔ cycle threshold (Ct) method [21].

**Table 1:** Primers used for the Real Time PCR study.

Gene	Primer Sequence (5′-3′)	Accession No.
GAPDH	F-CCTCTCTCTTGCTCTCAGTAT/R-GTATCCGTTGTGGATCTGACA	NM_017008.3
VEGF-A	f-Tgtgaatgcagaccaaagaaa/R-ctgaacaaggctcacagtgaat	AY702972.1
HGF	F-CCAGCTAGAAACAAAGACTTGAAAGA/R-GAAATGTTTAAGATCTGTTTGCGTT	NM_017017.2
bFGF	F-tcttcctgcgcatccatc/R-gcttggagctgtagtttgacg	X61697.1
IGF-1	F-atgcccaagactcagaagga/R-gtggcattttctgttcctc	X06043.1
TGF-alpha	F-gtattgtttccatgggacctg/R-cgtacccagagtggcagac	NM_012671.2

### TUNEL Assay

*In* *situ* detection of apoptosis was performed on heart tissue sections (5 µm) fixed in 4% paraformaldehyde using terminal deoxynucleotidetransferase (TdT) - mediated dUTP nick end labeling (TUNEL) kit (Roche, Germany) and positive cells were counted in five random fields. Relative TUNEL positivity was expressed as number of TUNEL positive cells/100 nuclei counterstained using Hoechst dye. Image-Pro plus 5.1 software was used for image capturing and cell counting (Media Cybernetics Inc., USA).

### Western Blotting

Heart tissues of saline treated and fC-MSC treated rats were homogenized in RIPA buffer containing 1 mmol/L phenylmethanesulphonyl fluoride (PMSF) and 1% protease inhibitor cocktail (Sigma-Aldrich, MO, USA). Tissue homogenate was centrifuge at 10000 rpm for 10 min and supernatant was stored. 40 mg proteins were loaded and separated by 10% SDS-PAGE. After electrophoresis, separated proteins were transferred to polyvinylidenedifluoride (PVDF) membranes. The membrane was blocked for 1 hour in 5% BSA at room temp followed by overnight incubation at 4°C with primary antibodies, Bax and Bcl-2 (Cell Signaling Technology, MA, USA). β- Actin antibody was used as an internal control. Primary antibodies were detected by corresponding horseradish peroxidase (HRP)-conjugated secondary antibodies using super signal west pico chemiluminescent substrate (Thermo scientific, IL, USA).

### Masson’s trichome Staining

Hearts tissues fixed in 10% formalin were cut into sections of 5 µm and stained with Masson’s Trichome stain (Glaxo Smith Kline, UK) to evaluate fibrosis.

### Statistical Analysis

Values were expressed as means ± SEM. Comparisons between saline and stem cell treated groups were made with the use of paired Student’s *t*-tests. *P*<0.05 was considered significant.

## Results

### Mesenchymal stem cell characteristics of fC-MSC

**Figure 1:**
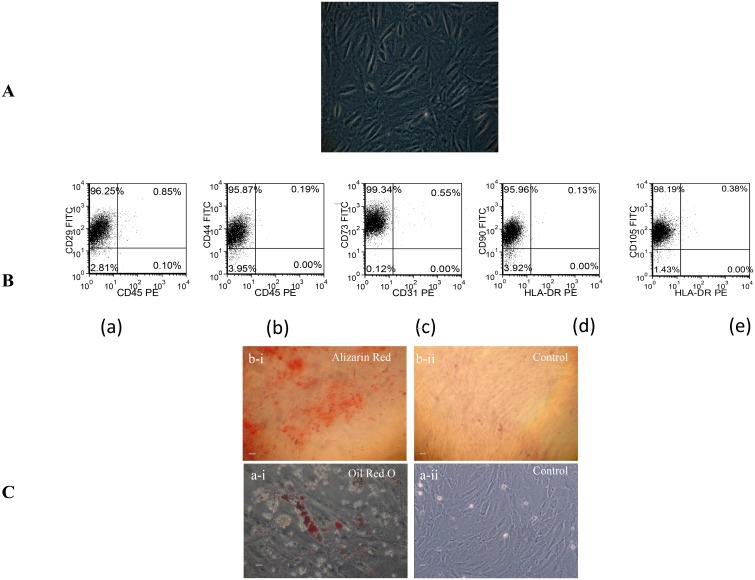
Morphology and characterization of fC-MSC (A) Representative photomicrograph (10X, 20 µm) of fC-MSC in culture showing spindle shaped morphology. (B) Representative flow cytometric dot-plots showing that fC-MSC are (a) CD29+/CD45−; (b) CD44+/CD45−; (c) CD73+/CD31−; (d) CD90+/HLA-DR−; (e) CD105+/HLA-DR−. (C) Representative photomicrographs (10X, 20 µm) showing differentiation of fC-MSC into Osteoblasts (a-i: differentiated cells positive for Alizarin red stain, and a-ii: control cells negative for Alizarin red stain) and Adipocytes (b-i: differentiated cells positive for oil red O stain, and b-ii: control cells negative for oil red O stain).

The fC-MSC grew as plastic adherent cells having trigonal or spindle shaped morphology from primary culture up to the 21^st^ passage. Flow cytometric analysis showed a typical mesenchymal phenotype of fC-MSC with expression of CD29 (96.54±0.44%), CD44 (95.96±0.10%), CD73 (95.10±0.16%), CD90 (98.90±0.18%), and CD105 (98.00±0.12%) and absence of CD31 (0.50±0.02%), CD45 (0.64±0.02%) and MHC-II (0.30±0.08%) markers. Treatment of fC-MSC with adipogenic and osteogenic induction media resulted in their differentiation into adipocytes and osteocytes as demonstrated by Oil red-O and Alizarin red staining, respectively (Fig. 1).

### Effect of fC-MSC on LV function and perfusion after MI

**Figure 2:**
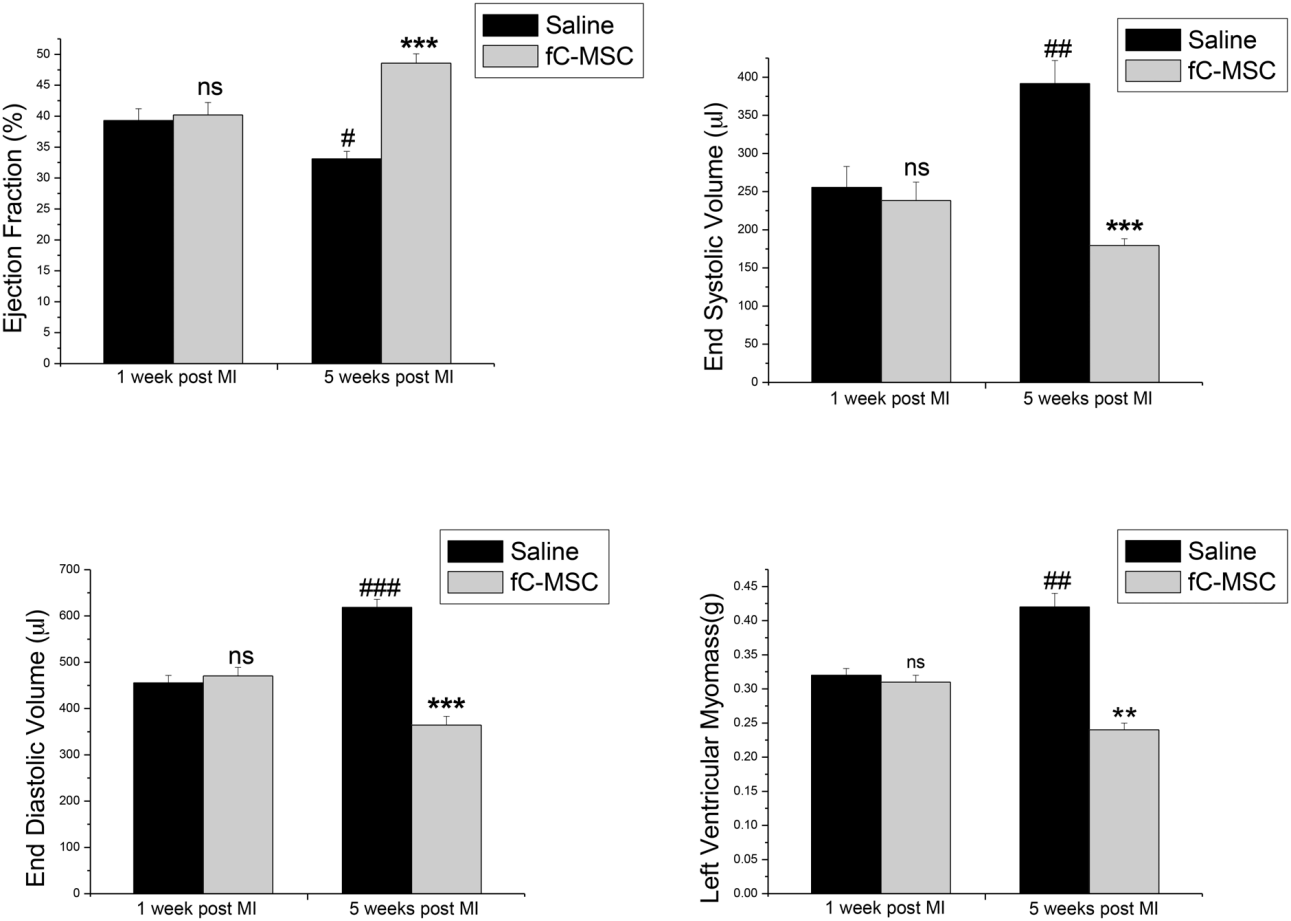
Effect of fC-MSC on LV functions: Bar diagrams showing ejection fraction, end systolic volume, end diastolic volume, and left ventricular myo-mass, measured at 1 week after MI (before fC-MSC therapy) and 4 weeks after fC-MSC therapy using gated SPECT analysis. Values shown are mean ± SEM (n = 6); *P<0.05, *P<0.01, *P<0.001 saline group after cell therapy vs before cell therapy (within the group);^ #^P<0.05,^ #^P<0.01, ^#^P<0.001 fC-MSC group after cell therapy vs before cell therapy (within the group);^ †^P<0.05, ^†^P<0.01, ^†^P<0.001 fC-MSC group (after cell therapy) vs saline group (after cell therapy).

Myocardial gated SPECT was used to measure LV function and cardiac perfusion 1 week after MI and 4 weeks after fC-MSC therapy. One week after MI, no significant differences in the ejection fraction (EF), end diastolic volume (EDV), end systolic volume (ESV) and left ventricular (LV) myo-mass were observed between saline and fC-MSC treated hearts (Table 2 and Fig. 2). After 4 weeks of cell/saline therapy, the fC-MSC treated group showed a significant increase in EF from the EF observed 1 week after MI and before cell therapy (48.57±1.5 vs 40.2±2, p<0.001), whereas EF remained significantly depressed in the saline treated animals (33.1±1.2 vs 39.3±1.9, p<0.01). Furthermore, there was a significant difference in EF between fC-MSC and saline treated groups, 4 weeks after cell/saline therapy (48.57±1.5 vs 33.1±1.2, p<0.001). Although both saline and fC-MSC treated hearts showed cardiac remodeling at 1 week after MI, the EDV, ESV and LV myo-mass of fC-MSC treated hearts showed a significant decrease at 4 weeks after therapy as compared to saline treated hearts (EDV 364.5±18 vs 618.5±17.6 µl, p<0.001; ESV 391.66±30.2 vs 179.5±8.7µl, p<0.001; LV myo-mass 0.24±0.01 vs 0.42±0.02 g, p<0.01) (Table 2 and Fig. 2).

**Table 2:** Gated SPECT analysis of fC-MSC therapy in rats with MI.

Groups	Ejection Fraction (%)	End Diastolic Volume (µl)	End Systolic Volume (µl)	LV Mass (g)
Healthy Control (Before MI)	59.83±1.19	261.3±22.11	104.66±8.88	0.21±0.008
MI baseline (1 Week after MI)	39.3±1.9***	455.6±16.1***	255.66±17.51**	0.32±0.01***
MI+ Saline (5 weeks post MI)	33.1±1.2^†^	618.5±17.6^†††^	391.66±30.2††	0.42±0.02††
MI+ fC-MSC (5 Weeks post MI)	48.57±1.5^‡‡^ §§§	364.5±18.7^‡‡^ §§§	179.5±8.7^‡‡^ §§§	0.24±0.01^‡‡^ §§

***P<0.001,

**P<0.01 = Healthy Control Vs MI Baseline;

†† P<0.01 = MI Baseline Vs MI+Saline;

‡‡‡ P<0.001 ^‡‡^P<0.01,

‡ P<0.05 = MI baseline Vs MI+fC-MSC;

§§§ P<0.001,

§§ P<0.01 = MI+Saline Vs MI+fC-MSC.

**Figure 3:**
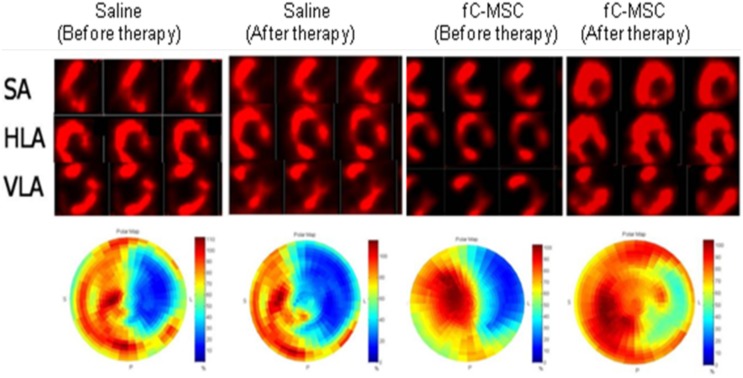
Effect of fC-MSC on LV perfusion: Representative SPECT perfusion images and polar-maps obtained at 1 week after MI (before fC-MSC therapy) and 4 weeks after fC-MSC therapy. (A) Serial 99mTc-sestamibi perfusion images obtained in SPECT short axis (SA), horizontal long axis (HLA), vertical long axis (VLA) of MI hearts treated with saline and fC-MSC (B) Corresponding polar-maps. The perfusion images (A) and the polar-maps (B) show a myocardial-flow defect in the anterolateral wall of left ventricle in both the groups. However, the hearts treated with fC-MSC demonstrate a smaller ischemic lesion (region of deficit) and a better perfusion in the MI segments.

The extent of ischemic region and its myocardial perfusion were evaluated at 4 weeks after fC-MSC administration. As can be seen in Fig. 3, the hearts treated with fC-MSC demonstrated a smaller ischemic lesion (region deficit, dark area) and a significantly greater 99mTc-sestamibi uptake than saline treated group (31.4±2.04% vs. 16.0±2.14%; p<0.001).

### *In vivo* tracking and engraftment of fC-MSC

**Figure 4:**
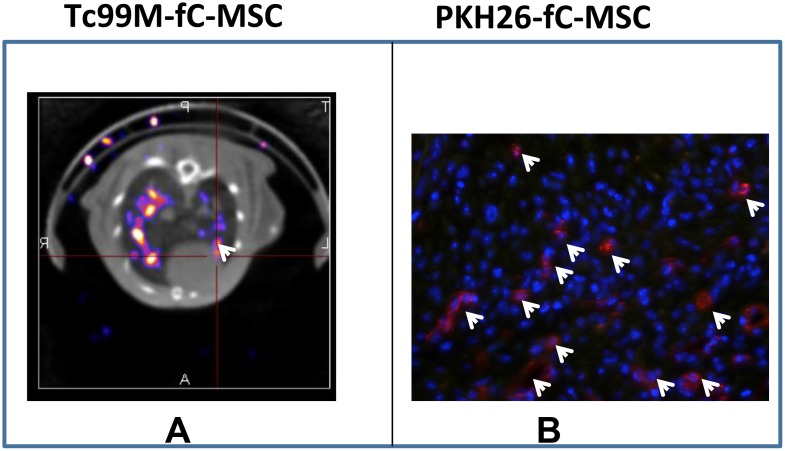
*In* *vivo* tracking and engraftment of fC-MSC: (A) Representative SPECT/CT images of fC-MSC treated hearts showing focal uptake of fC-MSC labelled with Tc-99 min the anterior mid-ventricular region of the heart6 hoursafter fC-MSC therapy. (B) Representative immunofluorescence photomicrographs of fC-MSC treated hearts showing PKH26 labelled fC-MSC in the injured hearts 4 weeks after fC-MSC therapy.

SPECT/CT imaging of MI rats 6 h after intravenous infusion of 99mTc-labeled fC-MSC revealed significant uptake of 99mTc-labeled cells in the lungs, with focal uptake in the anterior mid-ventricular region of the heart (Fig. 4A). Furthermore, to confirm the engraftment and retention of fC-MSC in follow up period, cells were labeled with PKH26. At 4 weeks of fC-MSC administration, cells labeled with PKH26 were located in the fibrotic region of the scar and more rarely in remote myocardium (Fig. 4B).

### Cardiomyogenic, endothelial and smooth muscle cell differentiation of fC-MSC *in vivo*

**Figure 5:**
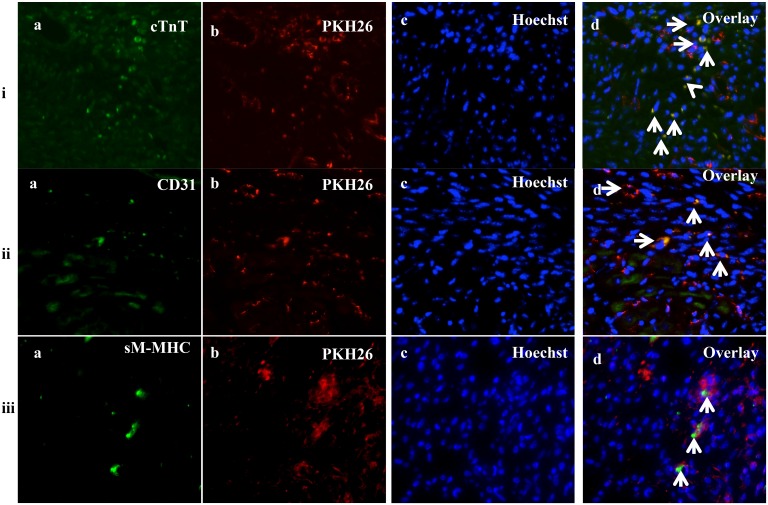
*In* *vivo* differentiation of fC-MSC in to cardiomyocytes, smooth muscle and endothelial cells. Representative immunofluorescence photomicrographs (40X; 20 µm) of fC-MSC differentiation into cardiovascular lineage cells observed in MI rat models 4 weeks after fC-MSC administration. Row (i): cardiomyocyte showing immunofluorescence staining for (a) Troponin-T; (b) PKH26 dye; (c) Hoechst dye; (d) Overlay of a, b & c images. Row (ii): endothelial cells showing immunofluorescence staining for (a) CD31; (b) PKH26 dye; (c) Hoechst dye; (d) Overlay of a, b & c images. Row (iii): smooth muscle cells showing immunofluorescence staining for (a) SM-MHC; (b) PKH26 dye; (c) Hoechst dye; (d) Overlay of a, b & c images.

Regenerating myocardial cells were detected by co-localization of immunostaining for cTnT (cardiomyocytes), sm-MHC (smooth muscle cells) and CD31 (endothelial cells) with PKH26 labelling. PKH 26 -labeled cells in the infarct/peri-infarct region also expressed cTnT, CD31, and sm-MHC indicating ability of fC-MSC to differentiate into cardiomyocytes, endothelial and smooth muscle cells (Fig. 5).

### Paracrine, anti-apoptotic and anti-fibrotic effects of fC-MSC

**Figure 6:**
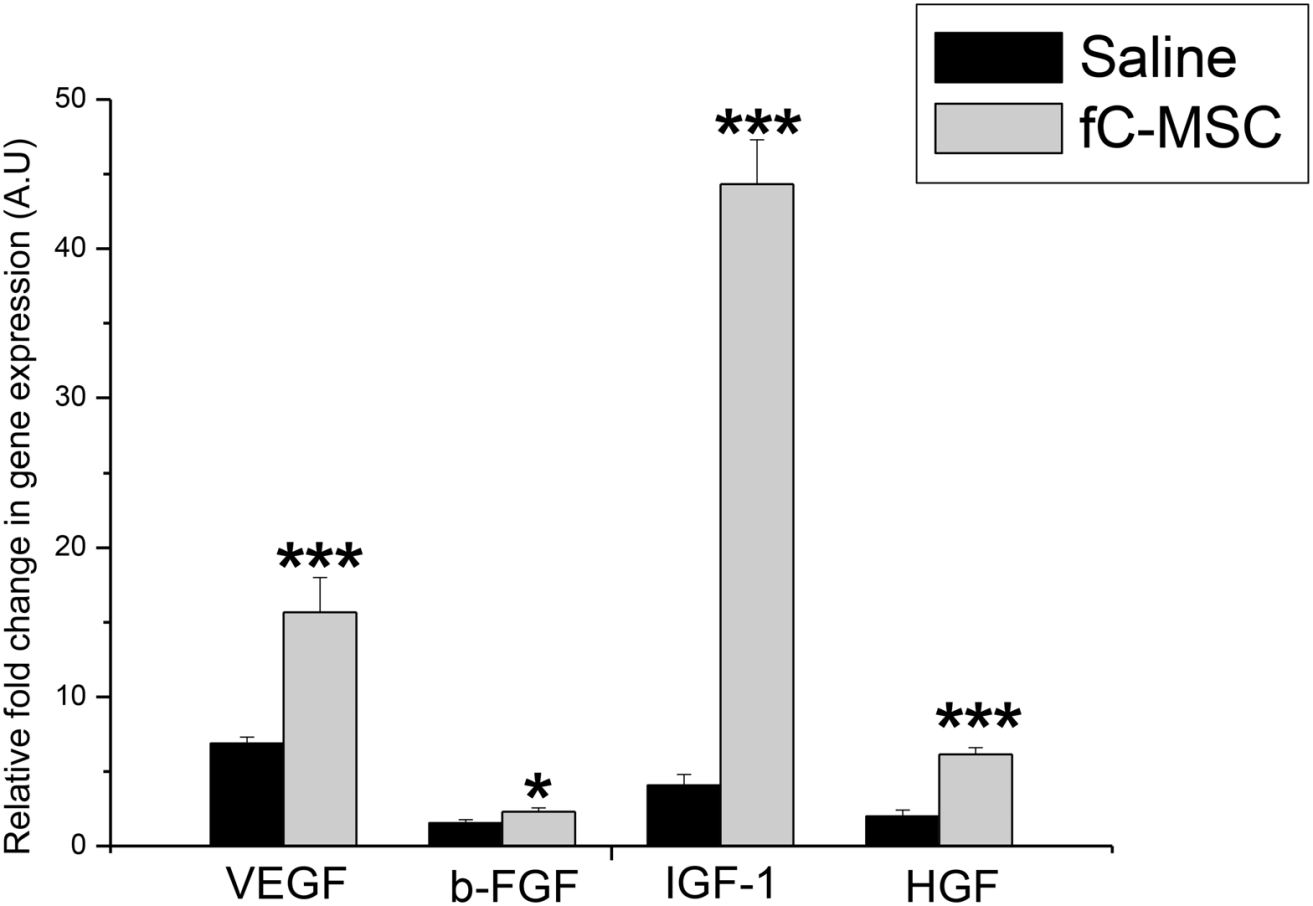
Effect of fC-MSC on gene expression of growth factors in rats with acute myocardial injury. Bar diagrams showing fold change expression of VEGF, b-FGF, IGF-1, and HGF in saline treated and fC-MSC treated ischemic hearts 4 weeks after fC-MSC administration. Values shown are mean ± SEM of 6 experiments, *P<0.05, **P<0.01, ***P<0.001: fC-MSC vs saline treatment.

**Figure 7:**
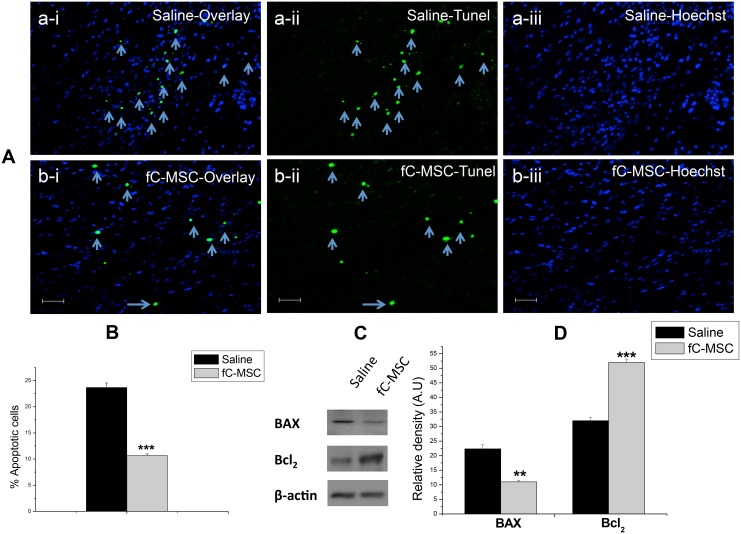
fC-MSC inhibit the apoptosis in rats with acute myocardial injury. (a) Representative immunofluorescence photomicrographs of saline and fC-MSC treated hearts 4 weeks after fC-MSC therapy showing (a-i & b-i) Overlay; (a-ii & b-ii) Tunel positive cells (green dye) counter stain with (a-iii & b-iii) Hoechst dye respectively (B) TUNEL apoptotic index showing significant decrease in apoptotic cells in fC-MSC treated compared to saline treated hearts. Values shown are mean ± SEM (n = 6). **P<0.01 fC-MSC treated vs saline treated hearts. (C) Representative immune-blots showing expression of BAX and BCL2 in saline and fC-MSC treated rats and (D) their relative density. Densitometric analysis was applied for comparison of relative protein expression. Values expressed Mean ± SE (n = 6), *P<0.05, **P<0.01, ***P<0.001: fC-MSC treated vs. saline treated group.

We screened hearts of MI rats for gene expression of growth factors by real time PCR and protein expression of pro/anti-apoptotic molecules by western blot. fC-MSC treated hearts showed significant up regulation in gene expression of various growth factors VEGF (40.2±0.9 vs 6.54±0.48 fold; p<0.001), βFGF (2.3±0.25 vs 1.3±0.1 fold, p<0.05), IGF-1 (61.6±4.4 vs 2.3±0.7 fold, p<0.001) and HGF-1 (5.18±0.5 vs 1.96±0.11 fold, p<0.001), compared to saline treated group (Fig. 6). We also observed a significant decrease in protein expression of pro-apoptotic molecule Bax (10.50±1.20 v/s 22.15±2.30, p<0.01) and increase in expression of anti-apoptotic molecule Bcl2 (52.75±2.57 v/s 33.4±1.75, p<0.001) in fC-MSC treated hearts as compared to saline treated hearts (Fig. 7A and B). TUNEL assay also revealed a significant decrease in apoptotic cells in fC-MSC treated group as compared to saline treated group (22±3 vs 11±2, p<0.001) (Fig. 7C and D).

**Figure 8:**
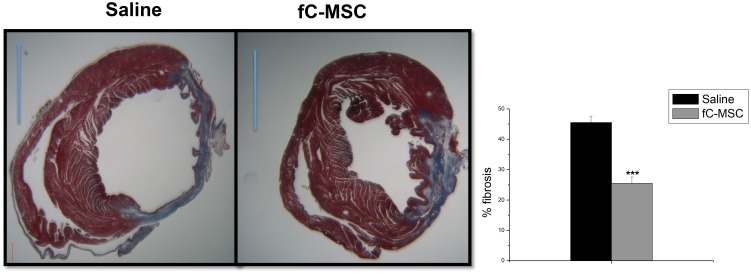
fC-MSC attenuate the myocardial fibrosis in rats with acute myocardial injury (A) Representative heart sections stained with Massons Trichome showing decrease in myocardial fibrosis in fC-MSC treated compared to saline treated hearts 4 weeks after fC-MSC administration. (B) Bar diagram showing percentage of fibrosis in saline treated and fC-MSC treated hearts. Values shown are mean ± SEM (n = 6). **P<0.05, **P<0.01, ***P<0.001: fC-MSC treated vs saline treated hearts.

Masson’s Trichome staining of tissue slices of MI hearts demonstrated an attenuation of myocardial fibrosis in the fC-MSC treated group, in comparison to the saline treated group (27±1.4 vs 12±1.15%, p<0.001; Fig. 8A and B).

## Discussion

The main objective of the present study was to determine the therapeutic capacity of fC-MSC as a novel stem cell type to treat myocardial infarction. We have demonstrated a significant improvement in the cardiac functions following fC-MSC therapy in a rat model of MI using the multi-pinhole SPECT/CT technology. We further demonstrated that intravenously administered fC-MSC were capable of engraftment in the ischemic myocardium and that the engrafted fC-MSC differentiated into all major cells of cardiovascular lineage. In addition, we also documented paracrine, anti-fibrotic and anti-apoptotic effects of fC-MSC.

We have isolated fC-MSC from rat fetal heart, which showed morphologic, phenotypic and differentiation characteristics similar to those of typical MSC. We recently reported that fC-MSC are capable of differentiation into all the three cell types of the cardiovascular tri-lineage over successive passages [14]. fC-MSC we identified also displayed cardiovascular transcription signature and could be induced to differentiate into all major cell types of cardiovascular lineage including cardiomyocytes, endothelial cells and smooth muscle cells *in* *vitro*. In addition these cells exhibited embryonal markers and extensive expansion potential in an undifferentiated state while maintaining expression of TERT and a normal karyotype. The tissue specific commitment and the primitive characteristics of fC-MSC together suggest their potential therapeutic value in cardiovascular regenerative medicine [14], [22]. Therefore to further explore the therapeutic effects of fC-MSC, we developed a rat model of cardiac ischemia-reperfusion (IR) injury by transient occlusion of descending coronary artery (LAD). Our model characteristically provides a large trans mural infarction extending from left ventricular apex to the lateral wall and recapitulates the phenotype of ischemic cardiomyopathy. Since acute inflammatory response immediately following reperfusion may result in clearance of administered cells from the infarcted region [23], we decided to inject fC-MSC only after this period of intense inflammation.

The success of any cell-based therapy for myocardial infarction injury is eventually moderated by the improvement of the cardiac functions. In this regard, echocardiography has been widely used to evaluate cardiac function after cell transplantation as it provides a safe, noninvasive, and inexpensive method. However, methods to image heart perfusion and *in* *vivo* tracking of stem cells in cardiovascular disease using small animal SPECT/CT is an exciting new area of research and has been less understudied. Therefore, in this study we performed multi pinhole 99m Tc-sestamibi gated SPECT/CT for non-invasive evaluation of cardiac perfusion and function in the rat heart at 1 week after MI and 4 weeks after the administration of fC-MSC. Moreover, this technology was also used for initial tracking of fC-MSC 6 h after administration. The systemic administration of fC-MSC attenuated post-infarction LV remodeling, as indicated by a significant improvement in heart function. The functional improvement observed with fC-MSC therapy was comparable with those observed with BMSC [24]–[26], and cardiac stem cells (CSC) [27], indicating that fC-MSC to be a potential novel cell types for cardiac repair. A recent study has shown adult heart cardiac stem cells expressing c-kit have an enhanced cardiopoietic potential compared to bone marrow-derived MSCs [28]. The same group has recently shown that co-transplantation of MSCs with CSCs reduces scar size and improves heart function post-MI, suggesting that combination therapy represent an effective and robust cell therapeutic strategy [29]. However, further studies to compare the therapeutic efficacy of C-MSC with other cell types are warranted to establish the superiority of fC-MSC over other cell types.

The initial tracking of the administered fC-MSC labeled with Tc99m revealed cells migration to the infarcted myocardium. Similar to our findings Barbash *et al* demonstrated cell migration to the heart in the first hours of systemic administration of MSC labeled with Tc99m in an open chest MI induced rat model. Because of short half-life of radionuclide Tc99m used for labeling of fC-MSC, the fC-MSC can only be tracked up to 6 hours following their injection [30]. To determine homing and retention of administered fC-MSC during follow up period, the cells were labeled with florescence dye, PKH-26. The preferential homing and retention of fC-MSC in the ischemic area of the myocardium might have contributed to efficient healing of the infracted heart. Although the underlying mechanisms remain unclear, ischemic tissue may express specific receptors or ligands to facilitate trafficking, adhesion, and infiltration of fC-MSC to ischemic sites. In the present study, some of engrafted fC-MSC expressed Troponin T, CD31 and sm-MHC, suggesting the potential of fC-MSC to differentiate into cardiomyocytes, endothelial and smooth muscle cells. fC-MSC represent a more primitive progenitor cell population than adult tissue-derived MSC and thus have the differentiation capability along cardiomyogenic lineages. These findings suggest that fC-MSC improved LV functions likely by increasing the local perfusion and also by myocardial regeneration.

Recent cell-based clinical studies suggest paracrine mechanisms for the observed changes in infarct remodeling or functional recovery. VEGF, HGF, and IGF-I have been shown to play important roles in cardiac repair [31]. Moreover, it has been recently reported that local administration of these growth factors in a porcine model of acute myocardial infarction resulted in reduction in the infarct size, increase in the capillary density and improvement in the cardiac contractile function [32]. In agreement with others [33], the present study also showed significant up-regulation in expression of these growth factors in infarcted rat hearts treated with fC-MSC compared to saline treated hearts. All these findings suggest that paracrine factors account for cardio-protective effects of fC-MSC therapy. Furthermore, fC-MSC were found to decrease fibrosis in ischemic myocardium as demonstrated by Masson’s Trichome staining. This result is consistent with the previous studies demonstrating that stem cell based therapies in ischemic myocardium improved cardiac function at least in part through an anti-fibrotic effect [34], [35]. In addition, we also found that fC-MSC protected cardiomyocyte from I/R induced apoptosis, as shown by a significant down-regulation in expression of pro-apoptotic molecule Bax and up-regulation in expression of anti-apoptotic molecule Bcl2 in infarcted heart following fC-MSC therapy. Detection of TUNEL positive cells also supports these findings. It has been shown that ratio of Bcl-2/Bax could be a key factor in the initiation of apoptosis through a mitochondrial-mediated pathway [36], Thus, fC-MSC may reduce injury through increasing ratio of Bcl-2/Bax.

In conclusion, we have demonstrated that fC-MSC engraft, differentiate into cardiac lineage cells and improve cardiac functions in rat model of MI. In addition, we demonstrate that paracrine, anti-fibrotic and ant-apoptotic effects of fC-MSC may be responsible for observed changes in post-infarct LV remodeling and functional recovery. Taken together, our results suggest that fC-MSC might hold substantial promise to develop a new therapeutic strategy for repair of myocardium after infarction. Finally, in acute myocardial infarction settings, pinhole gated SPECT/CT may be a useful tool to evaluate improvement in cardiac functions and track the cells fate after cell therapy.
